# Two-dimensional Mo_1.33_C MXene with divacancy ordering prepared from parent 3D laminate with in-plane chemical ordering

**DOI:** 10.1038/ncomms14949

**Published:** 2017-04-25

**Authors:** Quanzheng Tao, Martin Dahlqvist, Jun Lu, Sankalp Kota, Rahele Meshkian, Joseph Halim, Justinas Palisaitis, Lars Hultman, Michel W. Barsoum, Per O.Å. Persson, Johanna Rosen

**Affiliations:** 1 Thin Film Physics, Department of Physics, Chemistry and Biology (IFM), Linköping University, SE-581 83 Linköping, Sweden; 2 Department of Materials Science and Engineering, Drexel University, Philadelphia, Pennsylvania 19104, USA

## Abstract

The exploration of two-dimensional solids is an active area of materials discovery. Research in this area has given us structures spanning graphene to dichalcogenides, and more recently 2D transition metal carbides (MXenes). One of the challenges now is to master ordering within the atomic sheets. Herein, we present a top-down, high-yield, facile route for the controlled introduction of ordered divacancies in MXenes. By designing a parent 3D atomic laminate, (Mo_2/3_Sc_1/3_)_2_AlC, with in-plane chemical ordering, and by selectively etching the Al and Sc atoms, we show evidence for 2D Mo_1.33_C sheets with ordered metal divacancies and high electrical conductivities. At ∼1,100 F cm^−3^, this 2D material exhibits a 65% higher volumetric capacitance than its counterpart, Mo_2_C, with no vacancies, and one of the highest volumetric capacitance values ever reported, to the best of our knowledge. This structural design on the atomic scale may alter and expand the concept of property-tailoring of 2D materials.

## Abstract (web summary)

Vacancies in 2D materials can influence their properties, however controlling their formation remains a challenge. Here the authors show that selective etching of a 3D laminate with in-plane chemical ordering results in formation of MXenes with ordered divacancies, as well as elevated conductance and supercapacitance.

Two-dimensional (2D) ultrathin materials, for example, graphene, BN and MoS_2_, possess unique properties, which, can be utilized for various applications^1^,^2^,^3^. Defects, including vacancies, in these materials have been extensively studied, and shown to influence magnetic, electronic, catalytic and optoelectronic characteristics^4^,^5^,^6^,^7^,^8^. However, these defects are typically either unintentional or formed in macroscopic processes where the defect type and/or extent are not highly controlled. The prevailing challenge therefore is to master the order within the atomic sheets, on an atomic level, at a large scale.

One of the more promising applications of 2D solids is in the realm of energy storage, where intrinsically high specific surface areas and fast ionic diffusion through 2D channels result in high energy and power density electrodes. For example, previous reports on free-standing graphene-based electrodes^9^,^10^,^11^ demonstrate that combining large specific surface areas with good electrical conductivity has led to specific capacitances of up to 300 F cm^−3^. When electrically conductive 2D materials are capable of pseudocapacitive charge storage (that is, fast surface redox reactions)^12^, significantly higher energy and power densities can be achieved, as in the case of RuO_2_ (ref. 13) or Ti_3_C_2_ MXene^14^.

Discovered in 2011, MXenes are a comparatively young class of 2D materials obtained by the selective etching of A-group elements, mostly Al, from atomically laminated parent 3D M_*n*+1_AX_*n*_ (MAX) phases (where M is a transition metal, A is an A-group element, and X is C and/or N)^15^. When the A-element is etched, it is replaced by surface terminations, T_*x*_, and hence the proper MXene formula is M_*n*+1_X_*n*_T_*x*_, where T_*x*_ is a combination of –O, -OH and –F^16^. In many applications from energy storage^14^,^17^,^18^ to cationic adsorption^19^, conductive transparent electrodes^20^, and electromagnetic interference shielding^21^, MXenes perform as well as, if not better than, anything before them.

While it has long been appreciated that solid solutions can be formed on the M-, A- or X-sites, it is only quite recently that a quaternary MAX phase, Cr_2_TiAlC_2_, with out-of-plane chemical ordering was reported^22^. Several other out-of-plane MAX phases have since been discovered, and crucially the out-of-plane structure is preserved in their corresponding MXenes^23^,^24^. Herein we not only report on a type of ordered quaternary 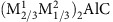 phase that we coin ‘i-MAX', wherein M^1^ and M^2^ are ordered in-plane, but also show how etching such solids results in 2D materials with ordered divacancies. A schematic of the MAX to MXene transformation is shown in Fig. 1a, where the divacancy configuration is best seen in the background schematic in Fig. 1a (right panel). We also report that this 2D material exhibits one of the highest conductivities of any MXene and possesses one of the highest specific capacitances, to the best of our knowledge. This introduction of ordered divacancies represents a breakthrough for property tuning of 2D materials which, critically, is also accompanied by a reduction in mass density.

## Results

### In-plane chemical ordering in a 3D MAX phase

**Figure 1:**
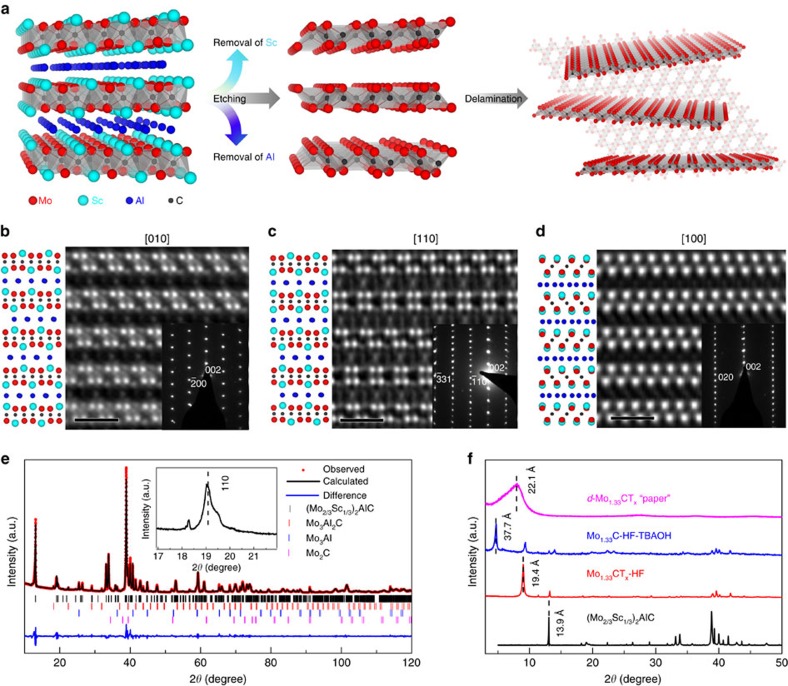
In-plane chemical ordering of (Mo_2/3_Sc_1/3_)_2_AlC leading to Mo_1.33_C MXene with ordered divacancies. (**a**) Schematic of (Mo_2/3_Sc_1/3_)_2_AlC before etching (left panel), after etching (middle panel) and after delamination (right panel). Background in the right panel shows the plane view of Mo_1.33_C MXene with ordered divacancies. (**b**–**d**) In-plane chemical ordering of the MAX phase evident from STEM images along the [010], [110] and [100] zone axis, respectively, with corresponding selected area electron diffraction (SAED). Schematics to the left of each image represent the corresponding atomic arrangements assuming the structure is the monoclinic space group *C2/c* (15). (**e**) Rietveld refinement of XRD of sample with nominal composition (Mo_2/3_Sc_1/3_)_2_AlC assuming same space group as above. (**f**) XRD pattern of (Mo_2/3_Sc_1/3_)_2_AlC before (black), after HF etching (red), and TBAOH intercalation (blue) and delamination (purple). Scale bars in (**b**–**d**) correspond to 1 nm.

The quaternary MAX (Mo_2/3_Sc_1/3_)_2_AlC with in-plane ordering, left panel in Fig. 1a, was discovered through the interplay between material synthesis and characterization followed by first-principles calculations based on Density Functional Theory (DFT) (see Methods section below). The calculations show that this phase is the most stable with respect to all competing phases in the Mo–Sc–Al–C system (Supplementary Table 1), and that the monoclinic (*C2*/*c*) and orthorhombic (*Cmcm*) structures are almost degenerate in energy (Supplementary Fig. 1 and Supplementary Table 2). Furthermore, from the calculated phonon dispersion spectra (Supplementary Fig. 2) we conclude that (Mo_2/3_Sc_1/3_)_2_AlC is also dynamically stable, that is, stable to lattice vibrations with no imaginary phonon frequencies.

Scanning transmission electron microscopy (STEM) images of the MAX phase along [010], [110] and [100] zone axes are shown, respectively, in Fig. 1b–d. In these micrographs, the Mo atoms appear brightest; the Sc and Al atoms are less bright. The schematics to the left of each micrograph sketch the atomic arrangements expected, assuming monoclinic (Mo_2/3_Sc_1/3_)_2_AlC. In Fig. 1d, the structure looks identical to a traditional MAX phase viewed along the [11–20] zone axis. However, evidence for in-plane ordering of the two transition metals is revealed in Fig. 1b,c, respectively, where the former image rules out orthorhombic symmetry (Supplementary Fig. 1). Most notably, the Sc atoms extend out of the Mo planes towards the Al layers. Consequently, the Al layers form a Kagomé lattice, evident from the bright–dark contrast variation in that layer. Lastly, the in-plane ordered structure is also revealed by the selective area electron diffraction shown in the insets of Fig. 1b–d.

Figure 1e shows the XRD patterns of a sample with nominal composition (Mo_2/3_Sc_1/3_)_2_AlC, for which the metal ratios are confirmed by energy-dispersive X-ray analysis (EDX). The Mo/Sc atomic ratio was found to be close to two (Supplementary Fig. 3). The Rietveld refinement based on space group *C2/c* (#15) is included in Fig. 1e, with the refinement parameters listed in Supplementary Table 3. Note that while the material before etching is not single phase, after etching it is (see below).

The lattice parameters calculated from the XRD Rietveld analysis, *a*=9.3486(1) Å, *b*=5.3985(1) Å and *c*=13.8738(2) Å, are consistent with theory and closer to those for the monoclinic than the orthorhombic structure (Supplementary Table 2). Note that the 110 peak—corresponding to an interplanar distance of 4.7 Å—appears in the XRD pattern around 19° (inset in Fig. 1e). This peak does not exist in the ternary 211 phases. This superstructure peak thus occurs solely due to Mo/Sc chemical ordering and is potentially quite useful for the identification of other in-plane ordered 211 MAX phases.

### Ordered divacancy formation in 2D MXene

Now that the ordered structure of the parent phase is established, we turn our attention to its MXene. Figure 1f plots the XRD patterns of (Mo_2/3_Sc_1/3_)_2_AlC powders, (i) before etching in hydrofluoric acid (ii) after etching in hydrofluoric acid (HF) and (iii) with subsequent intercalation with the organic base, tetrabutylammonium hydroxide (TBAOH). The corresponding *c* lattice parameters (LPs), expressed in hexagonal coordinates, are 13.9, 19.4 and 37.7 Å. The first is typical of the 211 MAX phases, the second typical of a 211 MXene^18^ and the third typical of a 211 MXene intercalated with TBAOH^25^. Note that the total disappearance of all but the (000*l*) MXene peaks implies that the entire sample was converted to MXene. The delaminated MXene will henceforth be referred to as *d*-Mo_1.33_C.

After TBAOH treatment, the MXene flakes delaminate spontaneously in water. The colloidal suspension, comprising of delaminated flakes, was filtered through a nanoporous polypropylene membrane to obtain free-standing *d*-Mo_1.33_C ‘paper' (Supplementary Fig. 6). XRD of this ‘paper' (Fig. 1f (topmost scan)) shows quite a broad asymmetric (0002) peak around 8°, which corresponds to a *c-LP* of 22.1 Å. EDX of the sample before, and after, etching (Supplementary Fig. 3) shows that both Al and Sc are absent after etching. Like all other MXenes, the Al layers are replaced by F and O-based terminations^16^. X-ray photoelectron spectroscopy (XPS) measurements on the *d*-Mo_1.33_C ‘paper' were performed for detailed identification and quantification of the terminating species (Supplementary Fig. 7 and Supplementary Table 4), giving a chemical formula of Mo_1.2_CO_0.7_(OH)_0.5_F_1.1_·0.4H_2_O_ads._ (using C as the base) with an error of <±0.2. Most interesting is that the material contains more –F terminations compared to the corresponding *d*-Mo_2_CT_*x*_ ‘paper', for which XPS gives the chemical formula Mo_2_C_0.9_O_1.0_(OH)_1.2_F_0.1_·0.4H_2_O_ads._ (using Mo as the base)^26^. The significant difference in surface terminations between the Mo-based MXene with and without vacancies will undoubtedly influence the material properties, which is a topic of future investigations. We also note in passing that (Mo_2/3_Sc_1/3_)_2_AlC can be etched by a combination of LiF and HCl (Supplementary Figs 3 and 4). Here again, after etching, the Al and Sc atoms are completely removed and replaced by F, O and some Cl.

**Figure 2:**
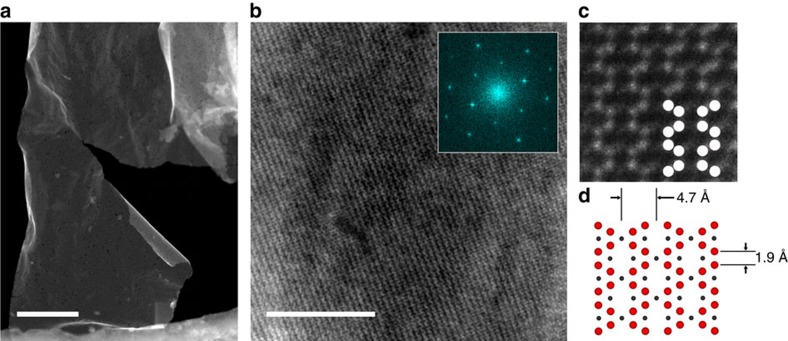
Top view of HAADF-STEM of single Mo_1.33_C sheet with ordered divacancies. (**a**) Low-magnification image of single flake with lateral dimensions >1 μm. (**b**) Higher magnification, with the FFT of the original image in (**a**) shown in the inset. (**c**) Atomically resolved image with overlaid schematic atomic structure in comparison to (**d**) ideal atomic structure from theoretically simulated parent MAX phase. The scale in **c**,**d** is identical. Scale bar in **a** corresponds 200 nm and scale bar in **b** corresponds to 10 nm.

An overview of the emerging monolayered structure can be seen in Fig. 2a, where a HAADF-STEM image of a single flake—with lateral dimensions >1 μm—is shown covering a hole in a holey carbon TEM grid. A medium magnification image and its Fast Fourier Transform (Fig. 2b) reveal a hexagonal-based crystal with chain-like features. The latter can be better seen in Fig. 2c, which clearly reveals the individual Mo atomic positions. When this micrograph is compared, side by side and at the same scale, with the one obtained from our DFT calculations (Fig. 2d) the agreement is excellent both qualitatively and quantitatively. In both cases, the undulating Mo-atomic chains are separated by ≈4.7 Å; the projected interatomic distance is ≈1.9 Å. The presence of divacancies results in a distinctive chain-like appearance, forming a sinusoidal pattern, which is mirrored in its neighbouring chain. In these micrographs the C atoms exhibit negligible contrast such that only the Mo atoms are imaged.

In 2015 we reported on a Mo_2_C MXene obtained by etching a MAX-phase-like material Mo_2_Ga_2_C (ref. 27). The Mo_1.33_C flakes synthesized here are equivalent to Mo_2_C, albeit with ordered vacancies in the position of every third Mo atom. Consequently, Mo_2_C and Mo_1.33_C allow a detailed exploration of the effect of divacancies on properties.

### Transport properties of Mo_1.33_C ‘paper'

At 33.7 μΩ m the room temperature (RT) resistivity of our 12 μm-thick *d*-Mo_1.33_C ‘paper' is about 4 orders of magnitude lower than that reported for a ∼9 μm-thick *d*-Mo_2_C ‘paper', namely 0.6 Ω m^26^. To the best of our knowledge, this is the highest-conductivity MXene ‘paper' ever reported. Furthermore, it should be noted that no efforts were made to remove the TBAOH between the layers that previous work has shown increases the resistance of Mo_2_C films^26^. The presence of vacancies has been shown to influence the conductivity in other materials, for instance, in TiO_2_ (ref. 28). Our finding that the resistivity is so much lower in the presence of vacancies is intriguing and is currently being explored.

### Electrochemical properties of Mo_1.33_C MXene

**Figure 3:**
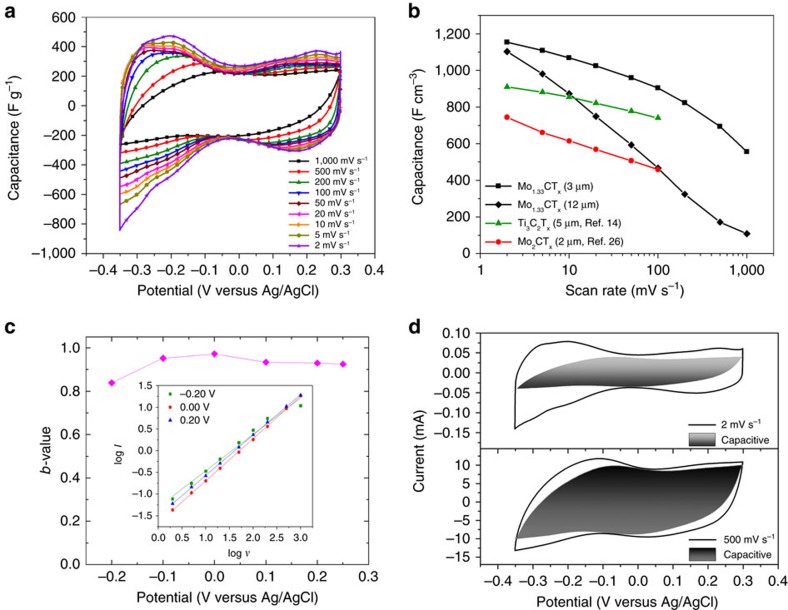
Electrochemical performance of Mo_1.33_C in 1 M H_2_SO_4_. Experiments were conducted in a three-electrode Swagelok cell. (**a**) Cyclic voltammograms of the 3 μm-thick electrode. (**b**) Scan rate dependence of specific capacitance of 3 and 12 μm thick free-standing electrodes. Also plotted are previous results on Ti_3_C_2_T_*x*_ clay^14^ and Mo_2_CT_*x*_^26^ (**c**) *b*-values for a 3 μm-thick film. Inset plots log *I* versus log *v* (see equation (1) in text). (**d**) CV partition analysis showing capacitive contribution to total current at select scan rates.

Previously, the capacitances of Ti_3_C_2_, Mo_2_TiC_2_ and Mo_2_C-based electrodes, in 1 M H_2_SO_4_ electrolyte, were reported to be quite high^14^,^24^,^26^. For comparison, our *d*-Mo_1.33_C ‘paper' electrodes were evaluated. When the cyclic voltammograms, CV, obtained at scan rates in the 2–1,000 mV s^−1^ range (Fig. 3a) are compared, it is clear that, at all scan rates, several broad peaks lead to large deviations from the rectangular voltammograms expected from double-layer capacitance alone. For example, at 2 mV s^−1^, broad anodic peaks (0.23, 0.11,−0.20, and 0.23 V versus Ag/AgCl) and cathodic peaks (0.16,−0.20, and −0.30 V versus Ag/AgCl) are present. Note that the voltammetric response of MoO_3_ electrodes tested in H_2_SO_4_ and other aqueous electrolytes^29^ also consist of broad peaks. These common features are not surprising given that Mo_1.33_C, like all other MXenes^14^,^26^, is terminated with –O, –OH and –F functional groups^16^.

Figure 3b plots, and Supplementary Table 5 lists, the scan rate dependencies of the specific capacitances. At 2 mV s^−1^, the volumetric capacitances—of a 3 μm-thick electrode—were 1,153 F cm^−3^ (339 F g^−1^). These record values are, respectively, ∼65% and ∼28% higher than those reported for a 2 μm-thick Mo_2_C paper (red circles in Fig. 3b)^26^ or a 5 μm-thick Ti_3_C_2_T_*x*_ electrode (green circles in Fig. 3b)^14^, respectively. At 1,000 mV s^−1^ capacitance values of 555 F cm^−3^ (163 F g^−1^) were still reached. For a thicker, 12 μm electrode, the capacitance values at 1,102 and 108 F cm^−3^ at 2 and 1,000 mV s^−1^, respectively, remain relatively high. Undoubtedly, the excellent electronic conductivity of this material and high density of ‘paper' electrodes (∼3.4 g cm^−3^) play an important role in these record capacitance values.

The results of galvanostatically charging the half cell at a current density of 10 A g^−1^ (Supplementary Fig. 8) reveal slight distortion from linear voltage profiles that would be expected for double-layer capacitance alone (inset), consistent with the CVs. After 10,000 charge/discharge cycles, 100% Coulombic efficiency and 84% of the initial capacitance were retained. The latter suggests that the high number of divacancies decreases the stability of Mo_1.33_C slightly in sulfuric acid, as compared to Mo_2_C MXene films, which when tested in the same way showed no loss in capacitance^26^.

## Discussion

To shed more light on the underlying physics, the CV data were analysed to characterize the extent of double-layer capacitive effects to the total charge stored assuming^30^:


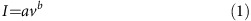


where *I* is the measured current at a given potential, *v* is the scan rate, and *a* and *b* are fitting parameters obtained from log *I* versus log *v* plots (inset in Fig. 3c). The resulting *b*-values (Fig. 3c) fall in the 0.84–0.97 range. Since *b*-values near 0.5 are indicative of diffusion-limited Faradaic intercalation processes, and those close to 1.0 are indicative of capacitive currents, the values obtained here suggest that, at all but the lowest potentials of −0.2 V versus Ag/AgCl, the measured current is capacitive in nature. Further quantification of the capacitive contribution to the total current was analysed using the Wang *et al*.^31^ approach. As shown in Fig. 3d, the capacitive contribution to the total current increases from 45% at 2 mV s^−1^ to 79% at 500 mV s^−1^. The high *b*-values, broad peaks in the CVs, and high specific capacitances observed suggest that pseudocapacitance is the major operative mechanism herein as postulated for other MXenes^14^,^24^,^32^,^33^. In the case of Ti_3_C_2_, direct evidence exists in terms of *in situ* X-ray absorption spectroscopy^32^. What is unclear at this time is whether the divacancies enhanced the capacitance values by increasing the electrochemically active sites and/or by increasing the fraction of the electrode accessible to the electrolyte. Clearly, more work, some of which is ongoing and beyond the scope of this paper, is needed to answer some of these fundamental questions.

To summarize, by selectively extracting both the Sc and Al atoms from a chemically in-plane ordered quaternary MAX phase (Mo_2/3_Sc_1/3_)_2_AlC, we realized a 2D Mo_1.33_C MXene with ordered divacancies. The latter, in turn, leads to higher conductivities and record volumetric capacitances for pure MXene films. It follows that an alternative path for property-tuning of 2D materials now exists. The implications and ramifications of this work are as follows: our 2D flakes with ordered vacancies can be readily made in aqueous environments, using a top-down approach, with yields that are close to 100%. The fact that the final flakes are less dense than their counterparts without vacancies is also noteworthy when specific properties are sought. We note in passing that the Sc used can easily be recycled. Based on the results shown herein, it is reasonable to believe that this approach may be of importance for energy storage and other applications.

## Methods

### Theoretical calculations

Electronic structure calculations were carried out using DFT using the projector augmented wave method^34^,^35^ with the Perdew–Burke–Ernzerhof (PBE)^36^ non-spin polarized generalized gradient approximation (GGA) exchange-correlation potential as implemented within the Vienna ab-initio simulation package (VASP)^37^,^38^,^39^. The electronic wave functions were expanded in a plane-wave basis set with a cutoff energy of 400 eV, and for sampling of the Brillouin zone we used Monkhorst–Pack *k*-point meshes to achieve a total energy convergence of less than 0.5 eV per atom^40^. Calculations are performed at zero temperature and pressure, and all structures were fully relaxed. To determine the dynamical stability of the studied ordered structure, we performed phonon calculations using finite displacement method along with Phonopy^41^.

### Synthesis of (Mo_2/3_Sc_1/3_)_2_AlC MAX phase

The powders used herein were graphite (99.999%), Mo (99.99%), (Sigma-Aldrich), Al (99.8% Alfa Aesar), and Sc (99.99% Stanford Advanced Material). To obtain the (Mo_2/3_Sc_1/3_)_2_AlC powder samples, a stoichiometric amount was mixed in an agate mortar, heated to 1,500 °C in an alumina crucible under flowing argon and held at that temperature for 20 h. After cooling down to RT in the furnace, a loosely packed powder was obtained. The powder was crushed and sieved through a 450 mesh sieve (particle size<32 μm). This fine powder was then used for XRD, EDX, STEM and MXene preparation.

### Synthesis of Mo_1.33_C MXene

One gram of the sieved powder was added to 20 ml 48% HF, stirring for 24 h at RT. After etching, the suspension was filtered, dispersed in water, and filtrated again. The washing procedure was repeated 5 times to remove any remaining acid and/or reaction products. The powder on the filtration membrane was dried at RT for 12 h. The dried powder was kept for further experiments and characterization. For exfoliation, 0.1 g of the etched powder and 1 ml TBAOH were added to a centrifuge tube, which was shaken manually for 5 min. The tube was then centrifuged at 6,000 r.p.m. for 5 min to remove the supernatant. Water was added to the tube to wash away the remaining TBAOH, after which the water was poured out. The process was repeated three times. The water was added carefully not to agitate the powder, in order not to delaminate the MXene. Finally, water was added to the powder and shaken for 5 min for delamination into single- or few- layered MXene. Another synthesis approach is to use LiF and HCl. Two gram of LiF was dissolved in 30 ml 12 M HCl. One gram of the sieved MAX powder was added to the LiF/HCl solution, stirring for 48 h at 35 °C. After etching, the suspension was filtered. Powder was added to a centrifuge tube and washed three times with 1 M HCl to remove excess LiF, and then three times with water. The HCl-washed slurry was thereafter washed three times with 1 M LiCl, and then three times with water. The supernatant was removed by centrifuging at 3,000 r.p.m. for 5 min using the above HCl and LiCl washing procedures. Finally, water was added to the washed slurry, and shaken for 5 min for delamination into single, or few, layered MXene.

### Materials characterization

XRD scans of the (Mo_2/3_Sc_1/3_)_2_AlC powders and *d*-Mo_1.33_C ‘paper' were carried out on a diffractometer (Panalytical X'pert). The XRD diffractogram of the (Mo_2/3_Sc_1/3_)_2_AlC powders was analysed by the Rietveld refinement method, using the *FULLPROF* code^42^,^43^. Refined parameters were: background parameters, scale factors, X and Y profile parameters for peak width, Lattice parameters, and atomic positions for all phases. The occupancies of Mo/Sc were refined manually by stepwise varying the ratio of Mo/Sc.

For the in-plane ordered MAX and MXene analyses, STEM combined with high angle-annular dark-field imaging (STEM-HAADF) and EDX analysis with a super-X EDX detector was performed in the double-corrected Linköping FEI Titan^3^ 60–300, operated at 300 and 60 kV, respectively. Selective area electron diffraction was performed on a FEI Tecnai T20 transmission electron microscope operated at 200 kV. Delaminated flakes were dispersed onto standard holey amorphous carbon support films suspended by Cu grids (SPI Supplies). Atomic resolution STEM-HAADF images were recorded using an optimized 30 mrad convergence angle, which provided Å-resolution probes at 60 kV, and 50 pA probe current. The HAADF detector's inner acceptance angle was set to 25 mrad.

XPS measurements were performed on a free-standing *d*-Mo_1.33_CT_*x*_ ‘paper' using monochromatic Al-K_α_ (1,486.6 eV) radiation in a Kratos AXIS Ultra^DLD^ system. The sample was mounted on a double-sided tape and grounded to the sample stage with copper contacts. Charge neutralization was performed using a co-axial, low-energy (∼0.1 eV) electron flood source to avoid shifts in the recorded binding energy, BE. XPS spectra were recorded for F 1s, O 1s, C 1s, Al 2p, and Mo 3d. The analyser pass energy used for all the regions was 20 eV with a step size of 0.1 eV. The BE scale of all XPS spectra was referenced to the Fermi edge, which was set to a BE of 0 eV. The peak fitting was carried out using CasaXPS Version 2.3.16 RP 1.6 as in ref. 16.

### Preparation of *d*-Mo_1.33_C ‘paper'

The Mo_1.33_C ‘paper' was prepared from *d*-Mo_1.33_C flakes obtained by HF etching of (Mo_2/3_Sc_1/3_)_2_AlC, followed by TBAOH intercalation as described above. After TBOAH treatment, the sediment was mixed with DI water (1 g Mo_1.33_C per 10 ml of water) and de-aerated using Ar gas, followed by sonication in an ice-cooled ultrasonic bath for 1 h. The mixture was then centrifuged for 1 h at 5,000 r.p.m., and the supernatant was collected. The latter was vacuum-filtered onto nanoporous polypropylene membranes (Celgard 3501, 0.064 μm pore size, Celgard LLC) in air. The *d*-Mo_1.33_C ‘paper' was easily peeled off from the membrane to obtain free-standing ≈12 μm and ≈3 μm thick ‘paper'.

### Transport measurements

A linear, four-point probe geometry was used to measure the RT resistivity of *d*-Mo_1.33_C ‘paper' using a Physical Property Measurement System (PPMS) (Quantum Design, San Diego). Gold wires were attached to the films using silver paint. The errors in the resistivity values are estimated to be ∼5%, corresponding to the uncertainty in sample thicknesses.

### Electrochemical testing

Mo_1.33_C ‘paper', ≈3 μm and ≈12 μm thick films were used without further preparation as working electrodes in a three-electrode configuration. YP-50 activated carbon (Kuraray, Japan) electrodes were prepared and used as overcapacitive counter electrodes, according to the procedures outlined in ref. 14: A stainless steel Swagelok half-cell was assembled with Mo_1.33_C ‘paper' on a gold foil current collector as the working electrode, overcapacitive activated carbon on a Pt foil current collector as the counter electrode and Ag/AgCl in 1 M KCl as the reference electrode. Electrochemical tests were conducted using a potentiostat/galvanostat (Bio-logic VMP3). CVs were obtained at scan rates in the 2 to 1,000 mV s^−1^ range after an initial pre-cycling of the cell 100 times at 20 mV s^−1^. Based on our experience, pre-cycling generally helps to saturate the interlayer space between the densely packed MXene sheets with electrolyte. The same half-cells were also galvanostatically charged and discharged for 10,000 cycles at a current density of 10 A g^−1^. The gravimetric capacitance values, *C*_m_, were calculated from cyclic voltammetry data assuming:


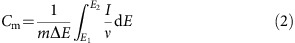


where *m* is the working electrode's mass, Δ*E* is the potential window, *E*_1_ and *E*_2_ are the potential limits, *I* is the measured current, and *ν* is the scan rate. Volumetric capacitance values were computed by multiplying the gravimetric capacitance by the working electrode density, which was 3.4 g cm^−3^.

### Data availability

All relevant data are available from the authors upon request.

## Additional information

**How to cite this article:** Tao, Q. *et al*. Two-dimensional Mo_1.33_C MXene with divacancy ordering prepared from parent 3D laminate with in-plane chemical ordering. *Nat. Commun.* **8,** 14949 doi: 10.1038/ncomms14949 (2017).

**Publisher's note**: Springer Nature remains neutral with regard to jurisdictional claims in published maps and institutional affiliations.

## Supplementary Material

Supplementary Information Supplementary Figures, Supplementary Tables and Supplementary References
