# Supplementary material for: Two-dimensional Mo1.33C MXene with divacancy ordering prepared from parent 3D laminate with in-plane chemical ordering
Source: Nat Commun. 2017 Apr 25;8:14949. doi: 10.1038/ncomms14949 (PMC5413966; doi:10.1038/ncomms14949)
Supplement: Supplementary Information — Supplementary Figures, Supplementary Tables and Supplementary References [file ncomms14949-s1.pdf]

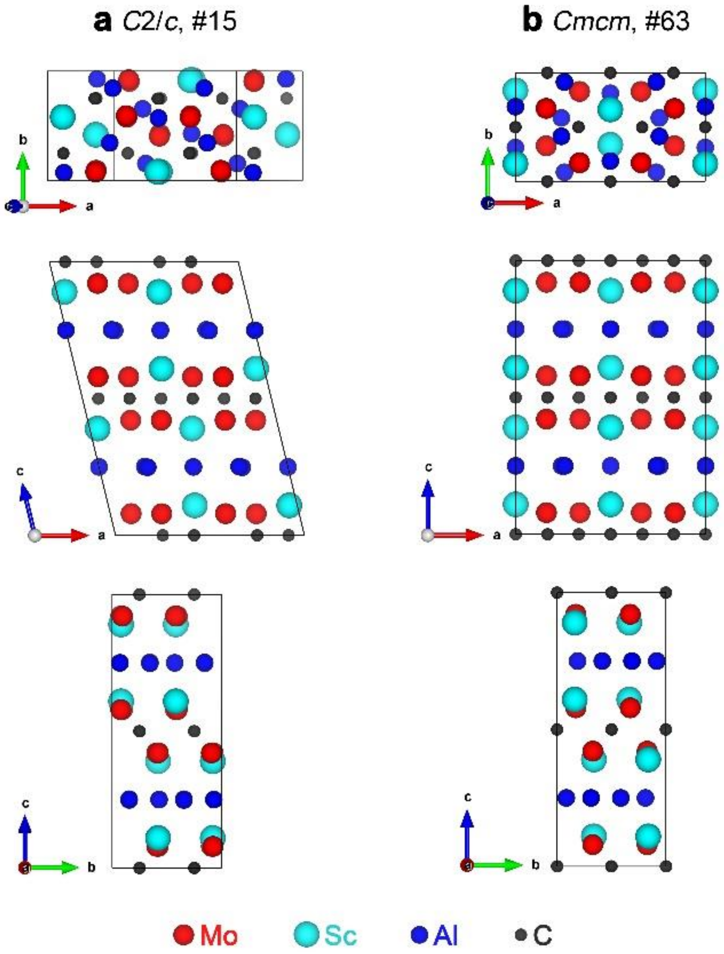

Supplementary Figure 1. Schematic representation of $(Mo_{2/3}Sc_{1/3})_2AlC$ assuming a, Monoclinic (*C2/c*) and b, orthorhombic (*Cmcm*) symmetry. Further details of these structures are given in Supplementary Table 2.

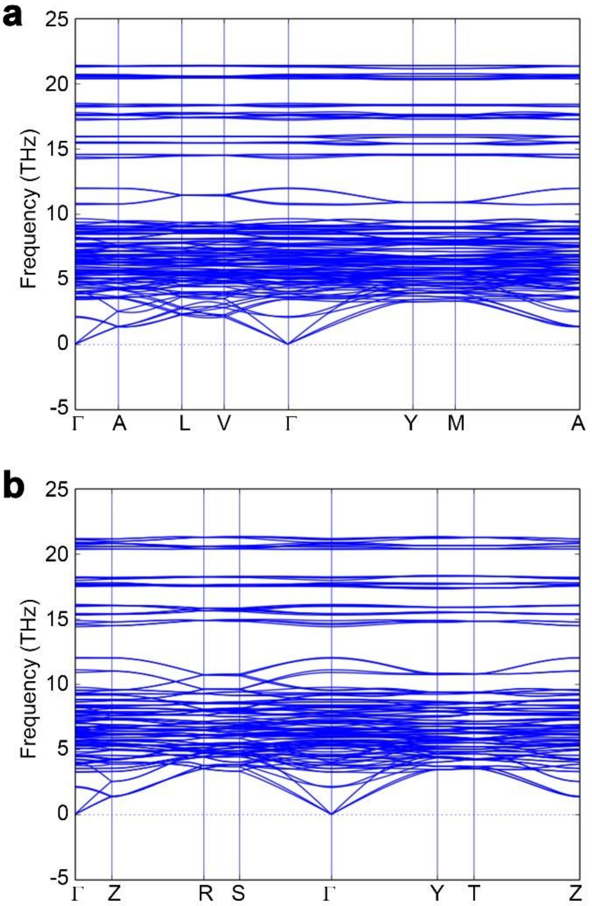

Supplementary Figure 2. Calculated phonon dispersion of $(Mo_{2/3}Sc_{1/3})_2AlC$. a, Monoclinic structure with space group *C2/c* (supercell size of $2\times2\times1$ unit cells). b, Orthorhombic structure with space group *Cmcm* (supercell size of $3\times3\times1$ unit cells.

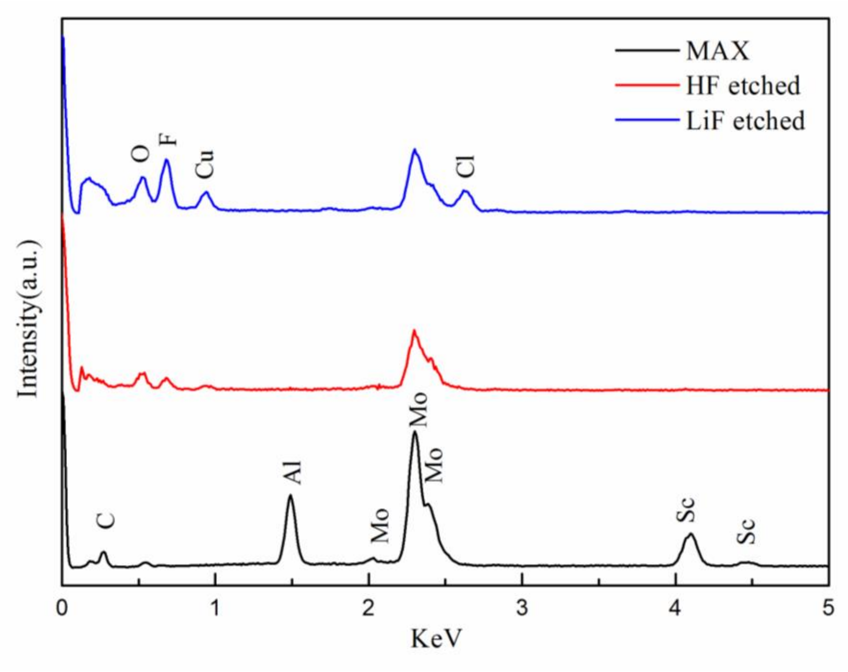

Supplementary Figure 3. EDX spectrum of $(Mo_{2/3}Sc_{1/3})_2AlC$ before and after etching. Spectrum taken in connection to TEM analysis, showing that both Al and Sc are absent after etching.

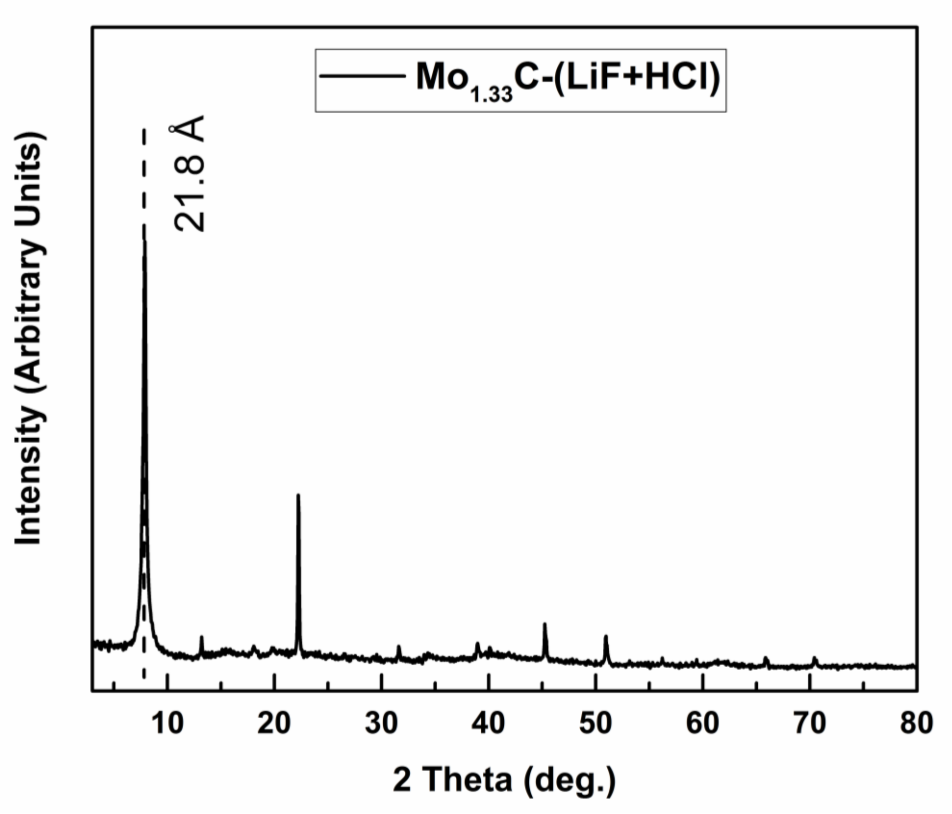

Supplementary Figure 4. XRD pattern for $(Mo_{2/3}Sc_{1/3})_2AlC$ etched with LiF+HCl. The c-LP of 22.37 Å is slightly larger compared to the one after HF etching (19.4 Å) presumably a result of Li-ion intercalation, as previously shown for Ti_3C_2 and Mo_2C MXenes[1, 2].

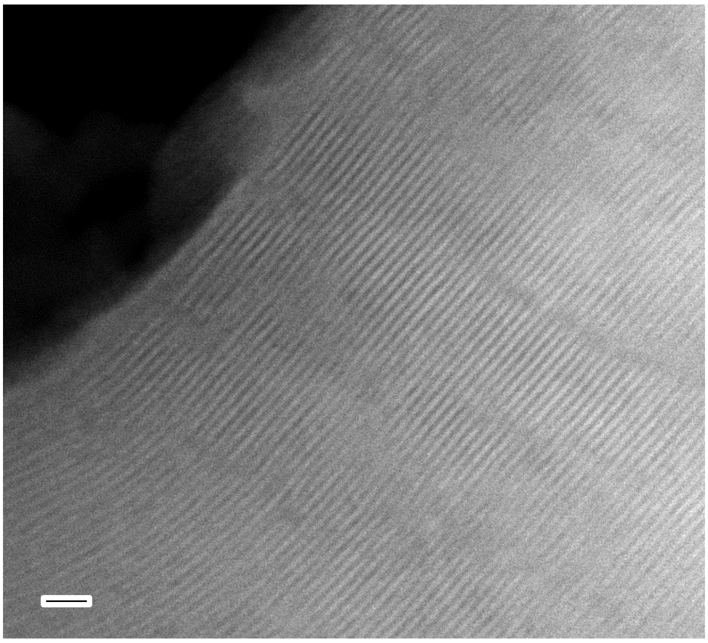

Supplementary Figure 5. Low magnification STEM showing side view of a multilayer $Mo_{1.33}C$ MXene flake. Scale bar corresponds to 10 µm.

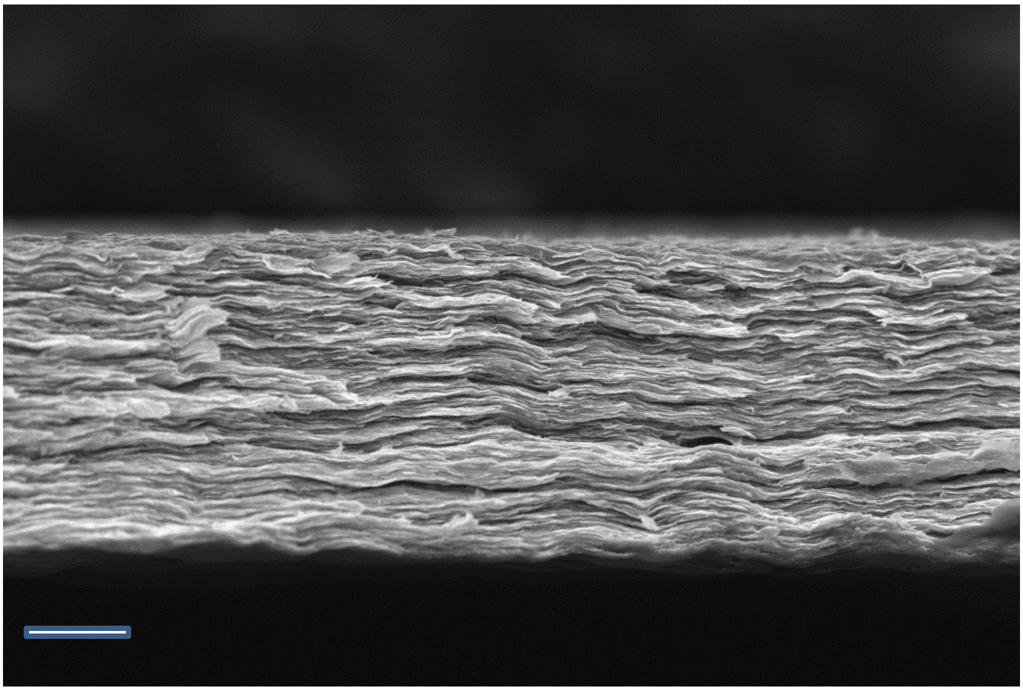

Supplementary Figure 6. SEM image showing cross section of the ≈ 3 µm thick *d*- $Mo_{1.33}C$ "paper". Scale bar corresponds to 1 µm.

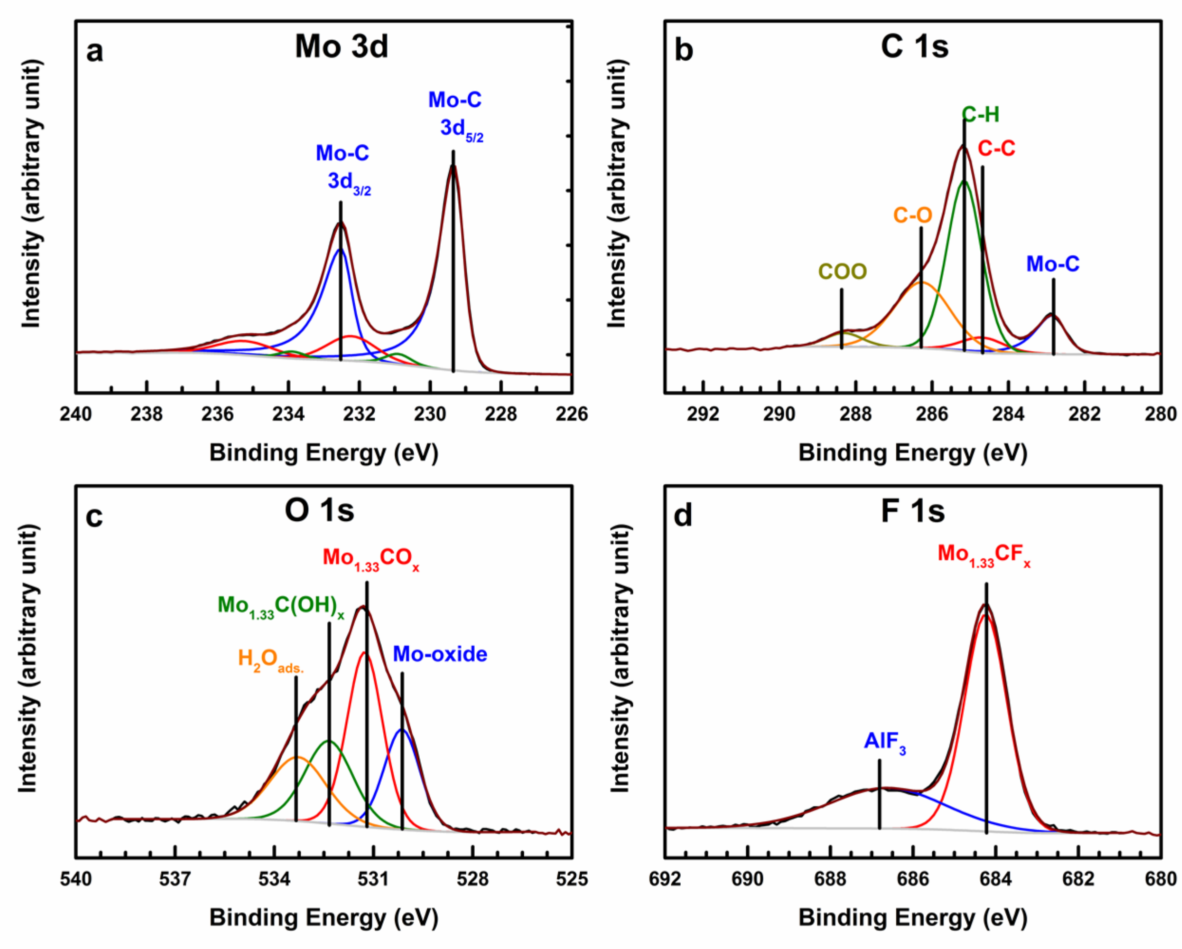

Supplementary Figure 7. High-resolution XPS spectra with peak fittings for **a,** Mo 3d **b,** C1s **c,** O1s and **d,** F 1s. The peak fitting results for various species and the resulting elemental compositions extracted from the high resolution spectra are tabulated in Supplementary Table 4.

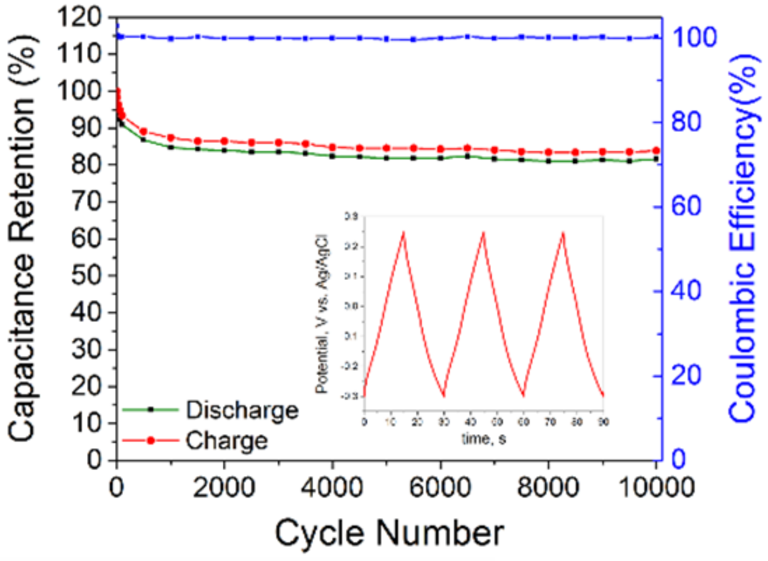

Supplementary Figure 8. Electrochemical performance of $Mo_{1.33}C$ in 1 M H_2SO_4 in a three-electrode Swagelok cell: Capacitance retention test on 3-μm thick $Mo_{1.33}C$ free-standing electrode at 10 A g^{-1}. Inset shows voltage vs. time profile for 3 cycles.

Supplementary Table 1.

Phases used in theoretical evaluation of phase stability in quaternary Sc-Mo-Al-C system. Last two entries are the most stable.

Phase	Prototype structure	Pearson symbol	Space group	V (Å^3/uc)	a (Å)	b (Å)	c (Å)	E_0 (eV/fu)
Sc	Mg	*hP*2	$P6_3/mmc$ (194)	49.25	3.321		5.157	-6.333
Sc	Sc	*hP*6	$P6_122$ (178)	148.75	3.242		16.342	-6.201
Sc	Np	*tP*4	P4/nmm (129)	100.35	5.367		3.484	-6.223
Mo	W	*cI*2	*Im*-3*m* (229)	15.92	3.169			-10.850
Mo	Cu	*cF*4	*Fm*-3*m* (225)	16.15	4.012			-10.431
Mo	Mg	*hP*2	$P6_3/mmc$ (194)	32.57	2.774		4.887	-10.414
Al	Cu	*cF*4	*Fm*-3*m* (225)	66.00	4.041			-3.745
Al	Mg	*hP*2	$P6_3/mmc$ (194)	33.28	2.856		4.712	-3.712
Al	W	*cI*2	*Im*-3*m* (229)	16.93	3.235			-3.649
C	C (graphite)	*hP*4	$P6_3/mmc$ (194)	38.14	2.464		7.250	-9.225
Al_4C_3	Al_4C_3	*hR*21	*R*-3*m* h (166)	245.00	3.355		25.129	-43.340
Sc_2Al	Ni_2In	*hP*6	$P6_3/mmc$ (194)	128.50	4.902		6.176	-17.458
ScAl	CsCl	*cP*2	*Pm*-3*m* (221)	38.75	3.384			-10.973
ScAl	CrB	*oC*8	*Cmcm* (63)	81.00	3.338	11.101	4.371	-10.892
$ScAl_2$	$MgCu_2$	*cF*24	*Fd*-3*m* (227)	109.50	3.797			-15.277
$ScAl_3$	$AuCu_3$	*cP*4	*Pm*-3*m* (221)	69.25	4.107			-19.383
$MoAl_{12}$	WAl_{12}	*cI*26	*Im*-3 (204)	436.23	7.584			-57.303
$MoAl_5$	$MoAl_5$	*hR*36	*R*-3*c* h (167)	558.49	4.952		26.296	-31.001
Mo_4Al_{17}	Mo_4Al_{17}	*mS*84	*C*2 (5)	1305.85	9.187	4.939	28.974	-112.563
Mo_3Al_8	Mo_3Al_8	*mS*22	*C*2/*m* (12)	334.46	9.235	3.653	10.091	-66.170
Mo_3Al	Cr_3Si	*cP*8	*Pm*-3*n* (223)	123.48	4.980			-37.228
Sc_2C	Ti_2C	*cF*48	*Fd*-3*m* (227)	852.33	9.481			-23.266
Sc_4C_3	P_4Th_3	*cI*28	*I*-43*d* (220)	188.75	7.227			-56.419
$ScC_{0.875}$	NaCl	*cF*8	*Fm*-3*m* (225)	208.70	4.708			-14.923
ScC	NaCl	*cF*8	*Fm*-3*m* (225)	25.70	4.685			-15.840
Sc_3C_4	Sc_3C_4	*tP*70	*P*4/*mnc* (128)	851.50	7.515		15.076	-58.764
MoC	TiP	*hP*8	$P6_3/mmc$ (194)	84.84	3.016		10.768	-19.821
MoC	NaCl	*cF*8	*Fm*-3*m* (225)	21.06	4.383			-19.640

MoC	η-MoC	*hp*12	*P*6$_3$/*mmc* (194)	126.16	3.074		15.401	-19.747
MoC	WC	*hp*2	*P*-6*m*2 (187)	21.00	2.928		2.829	-20.241
Mo_3C_2	Cr_3C_2	*oP*20	*Pnma* (62)	228.19	6.064	2.974	12.654	-50.938
Mo_2C	β''-Mo_2C	*hP*3	*P*-3*m*1 (164)	38.06	3.068		4.669	-31.064
Mo_3C	Fe_3C	*oP*16	*Pnma* (62)	215.87	5.540	7.559	5.159	-40.423
$ScAl_3C_3$	$ScAl_3C_3$	*hP*14	*P*6$_3$/*mmc* (194)	164.34	3.362		16.789	-47.703
Sc_3AlC	$CaTiO_3$	*cP*5	*Pm*-3*m* (221)	84.90	4.395			-35.023
Sc_2AlC	Cr_2AlC	*hP*8	*P*6$_3$/*mmc* (194)	141.75	3.296		15.065	-27.385
Sc_3AlC_2	Ti_3SiC_2	*hP*12	*P*6$_3$/*mmc* (194)	199.00	3.317		20.885	-43.406
Sc_4AlC_3	Ti_4AlN_3	*hP*16	*P*6$_3$/*mmc* (194)	248.50	3.296		26.414	-59.294
Mo_3AlC	$CaTiO_3$	*cP*5	*Pm*-3*m* (221)	71.70	4.154			-45.341
Mo_3Al_2C	Mo_3Al_2C	*cP*24	*P*4$_1$32 (213)	327.20	6.891			-50.299
$Mo_3Al_2C_{0.9375}$	Mo_3Al_2C	*cP*24	*P*4$_1$32 (213)	1303.30	6.881			-49.691
$Mo_3Al_2C_{0.875}$	Mo_3Al_2C	*cP*24	*P*4$_1$32 (213)	648.29	6.869			-49.078
$Mo_3Al_2C_{0.875}$	Mo_3Al_2C	*cP*24	*P*4$_1$32 (213)	1296.87	6.870			-49.069
$Mo_3Al_2C_{0.75}$	Mo_3Al_2C	*cP*24	*P*4$_1$32 (213)	321.10	6.848			-47.844
Mo_2AlC	Cr_2AlC	*hP*8	*P*6$_3$/*mmc* (194)	107.46	3.031		13.505	-35.292
Mo_3AlC_2	Ti_3SiC_2	*hP*12	*P*6$_3$/*mmc* (194)	151.49	3.072		18.541	-54.830
Mo_4AlC_3	Ti_4AlN_3	*hP*16	*P*6$_3$/*mmc* (194)	196.50	3.117		23.358	-74.552
$ScMo_2AlC_2$	$TiCr_2AlC_2$	*hP*12	*P*6$_3$/*mmc* (194)	154.85	3.062		19.072	-52.431
$MoSc_2AlC_2$	$TiCr_2AlC_2$	*hP*12	*P*6$_3$/*mmc* (194)	173.55	3.180		19.819	-48.262
$(Mo_{2/3}Sc_{1/3})_2AlC$	$(Mo_{2/3}Sc_{1/3})_2AlC$	*oP*48	*Cmcm* (63)	689.54	9.412	5.395	13.580	-99.925
$(Mo_{2/3}Sc_{1/3})_2AlC$	$(Mo_{2/3}Sc_{1/3})_2AlC$	*mS*48	*C*2/*c* (15)	689.78	9.367	5.427	13.961	-99.917

Supplementary Table 2

Atomic structure and phase stability of $(Mo_{2/3}Sc_{1/3})_2AlC$ assuming monoclinic (*C2/c*) or orthorhombic (*Cmcm*) symmetry. Last row lists the phase stability of each structure expressed in formation enthalpy ΔH_{cp} calculated with respect to the identified set of the most competing phases, viz. $ScMo_2AlC_2$, Mo_3Al, Sc_3AlC and Mo_3Al_8.

Space group	*C*2/*c* (#15)	*Cmcm* (#63)
Z	4	4
a (Å)	9.367	9.412
b (Å)	5.427	5.395
c (Å)	13.961	13.580
α	90	90
β	103.587	90
γ	90	90
Sc	8f (-0.04219, 0.41875, 0.10951)	8f (0.00, 0.16259, 0.60949)
Mo	8f (0.27241, 0.42159, 0.07902)<br>8f (0.61047, 0.40637, 0.07930)	16h (0.16195, 0.32801, 0.07925)
Al	8f (0.74256, 0.15523, 0.25135)<br>4e (0.00000 -0.07121 0.25000)	4c (0.00000, 0.31203, 0.25)<br>8g (0.25811, 0.08498, 0.25)
C	8f (0.41614 0.25145 0.00010)<br>4d (0.25, 0.25, 0.50)	8e (0.16755, 0.0, 0.0)<br>4b (0.0, 0.5, 0.0)
ΔH_{cp} (meV/atom)	-40.3	-40.9

Supplementary Table 3

Rietveld refinement of $(Mo_{2/3}Sc_{1/3})_2AlC$ assuming monoclinic (*C2/c*) symmetry. From the Rietveld refinement of the XRD pattern shown in Fig. 1E the mass fractions of the different phases were: $(Mo_{2/3}Sc_{1/3})_2AlC$ (84.9(5) wt.%), Mo_3Al_2C (7.5(1) wt.%), Mo_3Al (7.1(1) wt.%) and Mo_2C (0.5(1) wt.%). The total χ^2 value was 32.

Space group	*C2/c (#15)*
a (Å)	*9.3486(1)*
b (Å)	*5.3985(1)*
c (Å)	*13.8738(2)*
α	*90.0000*
β	*103.2288(10)*
γ	*90.0000*
Sc	*8f (0.9497(8) 0.4162(17) 0.0896(3))*<br>*Occupancy of Sc = 7.00 and Mo = 1.00*
Mo	*8f (0.2760(3) 0.4223(10) 0.0842(1))*<br>*Occupancy of Mo = 7.00 and Sc = 1.00*<br><br>*8f (0.6102(4) 0.4070(10) 0.0829(1))*<br>*Occupancy of Mo = 7.00 and Sc = 1.00*
Al	*8f (0.7562(9) 0.1533(19) 0.2547(7))*<br>*4e (0.00000 0.9671(20) 0.25000)*
C	*8f (0.4161 0.2515 0.0000)*<br>*4e (0.2500 0.2500 0.5000)*

Supplementary Table 4

XPS peak fitting of results shown in Supplementary Fig. 7 for *d*-$Mo_{1.33}CT_x$ "paper". The numbers in brackets in column 2 and 3 are peak locations of Mo $3d_{3/2}$ and their full-widths at half maximum, FWHM, respectively. For peak assignment see Ref. 2 and Ref. 3 and references therein.

Region	BE [eV][a]	FWHM [eV]	Fraction	Assigned to
Mo $3d_{5/2}$ ($3d_{3/2}$)	229.3 (232.5)	0.6 (0.7)	0.82	C-Mo-T_x
	230.9(233.9)	0.8 (0.8)	0.03	Mo^{+5}
	232.2 (235.2)	1.6 (1.9)	0.15	MoO_3
C 1s	282.8	0.8	0.11	C-Mo-T_x
	284.7	1.3	0.05	C-C
	285.2	1.1	0.49	CH_x
	286.3	1.7	0.31	C-O
	288.3	1.1	0.04	COO
O 1s	530.1	1.3	0.21	MoO_3
	531.3	1.3	0.36	C-Mo-O_x
	532.3	1.7	0.23	C-Mo-$(OH)_x$
	533.3	2.0	0.21	H_2O_{ads}
F 1s	684.2	1.2	0.66	C-Mo-F_x
	686.6	3.3	0.34	AlF_3

[a] Values in parenthesis correspond to the $3d_{3/2}$ component.

Supplementary Table 5

Gravimetric capacitance values for two different thickness *d*-$Mo_{1.33}C$ free-standing electrodes tested in a three-electrode Swagelok configuration in 1 M H_2SO_4.

	**3 µm-thick film**		**12 µm-thick film**	
	Gravimetric(F g^{-1})	Volumetric (F cm^{-3})	Gravimetric (F g^{-1})	Volumetric (F cm^{-3})
**2 mV s^{-1}**	339	1153	324	1102
**5 mV s^{-1}**	325	1107	288	980
**10 mV s^{-1}**	314	1068	256	872
**20 mV s^{-1}**	301	1024	220	749
**50 mV s^{-1}**	282	958	174	593
**100 mV s^{-1}**	265	903	137	466
**200 mV s^{-1}**	241	822	95	323
**500 mV s^{-1}**	204	693	50	171
**1000 mV s^{-1}**	163	555	32	108

Supplementary References

1. Ghidiu, M. *et al.* Ion-Exchange and Cation Solvation Reactions in Ti_3C_2 MXene. *Chem. Mater.* **28,** 3507-3514 (2016).
2. Halim, J. *et al.* Synthesis and characterization of 2D molybdenum carbide (MXene). Adv. Func. Mater. **26,** 3118-3127 (2016).
3. Halim, J. *et al.* X-ray photoelectron spectroscopy of select multi-layered transition metal carbides (MXenes). *App. Surf. Sci.* **362,** 406-417 (2016).
